# Marriage Lengthens Life

**Published:** 1872-05

**Authors:** 


### MARRIAGE LENGTHENS LIFE.

In France, Belgium, and Holland, six married men out of every thousand die in a year, of bachelors ten, and twenty-two of widowers, all between the ages of twenty-five and thirty years. Thus, —

| | | | |
|---|---|---|---|
| 25 to 30 years, | married 6, | celibates 10, | widowers 22 |
| 30 " 35 " | " 7 | " 11, | " 18 |
| 35 " 40 " | " 7 | " 13, | " 18 |

and so of all ages the married man has the better chance of a longer life.

The great mortality among widowers, far above the celibates, is the result of combined causes: —

First, A man never survives the memories of his first young love, early cut off.

Second, The responsibilities and cares connected with children, causing an incessant weight upon the mind, robbing it of its elasticity, and allowing it to pine away in vain regrets and unavailing sorrow.

### TOO EARLY MARRIAGES.

A terrible lesson is taught by these dry statistics; of eight thousand persons marrying before twenty, fifty-five die in one year, while only seven die under twenty who are not married; so persons from eighteen to twenty who do not marry have nine times more chance of living than those who do marry before twenty. The fact is that everywhere those who marry before twenty die as fast as persons from sixty to seventy years of age.

The reason is this: early marriage uses up the vital energies in marital indulgences and excesses, leaving the system many fold more liable to diseases of all kinds from slighter causes, and less able to withstand their inroads.

### THE LADIES

do not bear single life any better than gentlemen. From the age of thirty to thirty-five only nine married in a thousand die annually, eleven celibate; these proportions increase up to fifty-five years; for at fifty-five, a thousand wives show only fifteen deaths, but old maids twenty-five; out of a thousand unmarried girls from fifteen to twenty, only eight die annually; if married at that early age, twelve.

Where there are a hundred criminals of single men, there are but fifty of married; and it is a striking fact that widows and widowers are as likely to commit crime as those who have never been married, while the married are not half as likely to kill themselves as the unmarried.

From these statements the general truth presents itself, that marriage, not earlier than twenty-one, as to both males and females, promotes health, longevity, and morality.

